# Small bowel perforation after low-velocity trauma: a case report

**DOI:** 10.1093/jscr/rjab095

**Published:** 2021-04-19

**Authors:** S Sinz, L Benigno, M A Zadnikar, M E Biraima-Steinemann

**Affiliations:** Department of General, Visceral, Endocrine and Transplantation Surgery, Kantonsspital St. Gallen, Saint Gallen, Switzerland

## Abstract

We report the case of a 63-year-old patient with a low-velocity abdominal trauma and bowel perforation. The patient slipped on a wet floor and fell down the stairs. On admission, the patient complained about abdominal pain. A computed tomography scan showed traumatic hematoma of the jejunum in the left upper quadrant and a small amount of intra-abdominal air. Also rib fractures on the left side were diagnosed. We performed a diagnostic laparoscopy and found a bowel perforation, which was manually repaired.

## INTRODUCTION

Abdominal trauma is the fourth most common trauma mechanism in the adult. It is associated with high morbidity and mortality. According to a recent publication, the overall mortality rate is 8–25% [1]. The trauma mechanisms normally refer to car accident, gunshot wound or fall from height. Depending on the trauma mechanism, it is important to differentiate between sharp and blunt abdominal trauma. The prevalence of intra-abdominal injury among patients with blunt abdominal trauma is ~13% [2].

Splenic or hepatic lacerations are common sequelae of blunt abdominal trauma. However, bowel perforation is with 10% rather uncommon. To cause severe bowel injury, a high-velocity impact is necessary.

Here, we report a rare case of a low-velocity accident due to a fall down the stairs with blunt abdominal trauma and laceration of the small intestine.

## CASE REPORT

A 63-year-old patient was admitted to the hospital by ambulance after a fall down the stairs of about six steps after slipping on a wet floor at work. She fell on the left side of her body. There was no head impact or unconsciousness. The patient’s medical history was unremarkable; only a laparoscopic cholecystectomy was performed about 25 years ago.

On arrival, the patient was hemodynamically stabile, with guarding on the left hemiabdomen.

The blood levels on admission showed elevated lactate dehydrogenase (338 U/l), creatine kinase (701 U/l) as well as mild leucocytosis (12.6 G/l). The clinical examination showed tenderness on the left lower thorax with normal auscultation. The abdomen was tense with rebound tenderness in the left hemiabdomen. There was no bruising.

**Figure 1:**
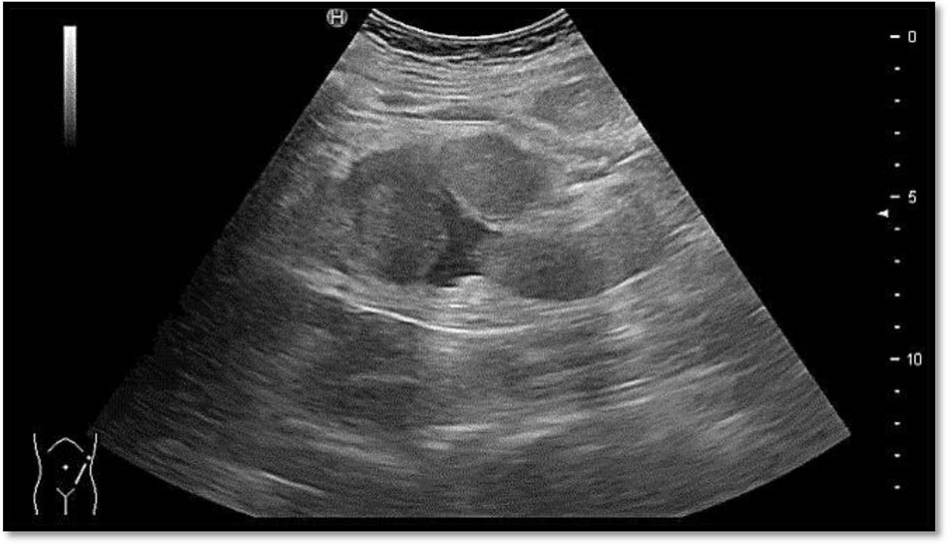
Abdominal sonography in the emergency department with free fluid on the left hemiabdomen.

The ultrasound revealed some trace amount of free fluid in the left part of the abdomen (Fig. 1).

**Figure 2:**
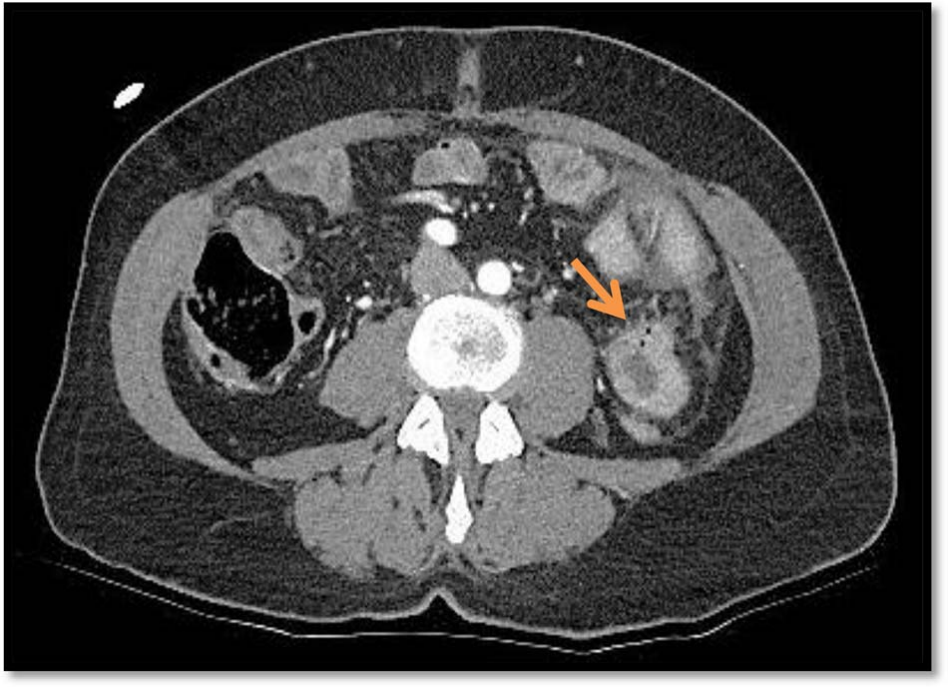
Free air bubbles on the CT scan in the emergency department.

Due to the unclear sonography findings, a computed tomography (CT) scan was performed, which was concerning for a bowel perforation and traumatic hematoma of the jejunum in the left upper quadrant (Fig. 2). Also rib fractures of left costae VII, IX–XII were detected.

**Figure 3:**
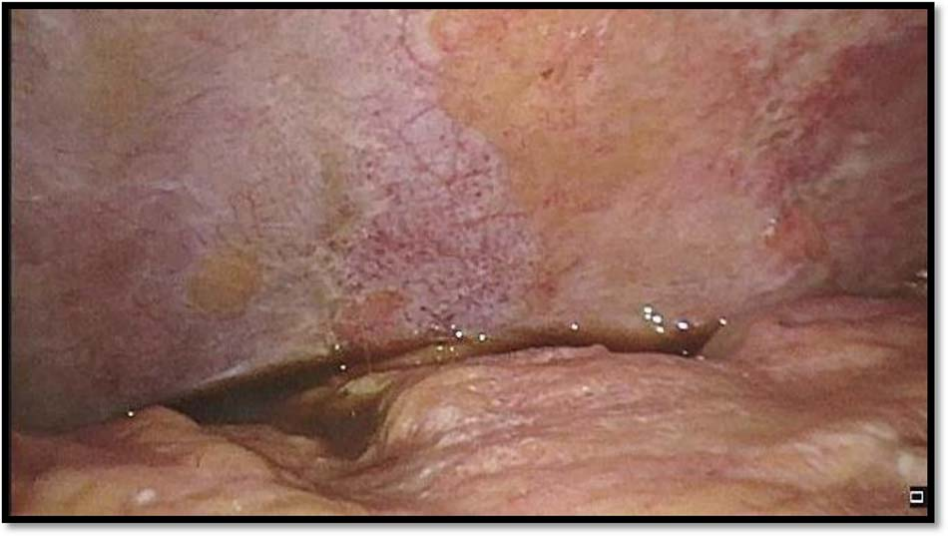
Left upper quadrant during diagnostic laparoscopy.

Considering the clinical presentation and the CT scan, we performed an emergency diagnostic laparoscopy. On the overview laparoscopy, we found free fluid in the left hemiabdomen as well as signs of moderate peritonitis (Fig. 3).

**Figure 4:**
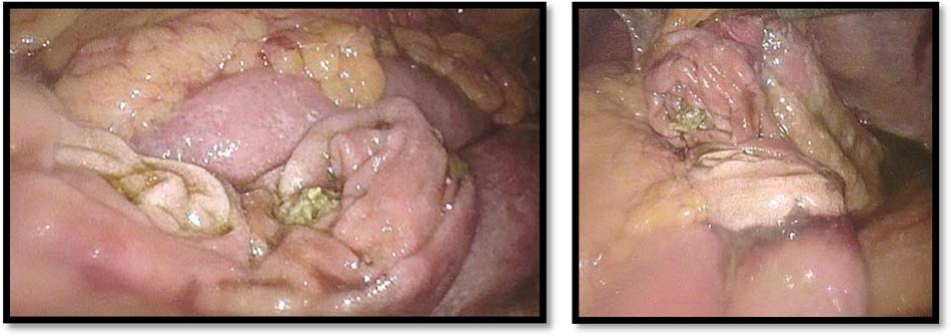
Nearby total circumferential perforation of the small intestine.

On inspection, we found a sharply demarcated, near circumferential perforation of the small intestine (Fig. 4).

**Figure 5:**
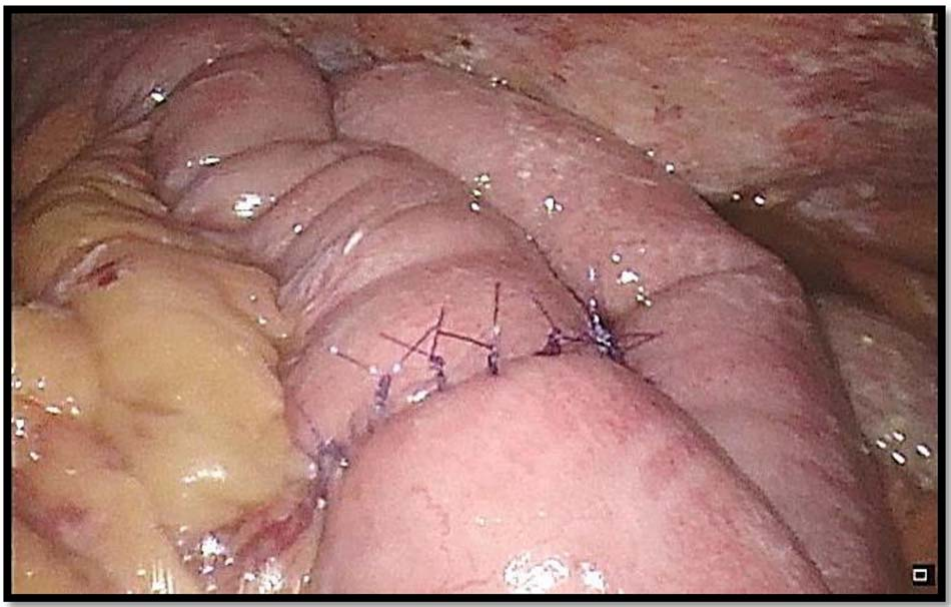
Re-anastomosis of small intestine.

We converted to a mini laparotomy and performed a handsewn primary repair of the small intestine (Fig. 5). The abdomen was washed and drains were placed.

The recovery of the patient was uneventful; a bowel movement was established on day 3. The oral food intake was well tolerated. The drains were removed after a few days. We discharged the patient on postoperative day 11.

## DISCUSSION

Traumatic blunt small intestinal perforation is a rare traumatic injury, especially in low-velocity trauma.

The mechanism of this straight bowel laceration is suggestive of a laceration by a broken rib. Otherwise, a mechanical stretch of the bowel over a fulcrum (e.g. the spine) is also possible. In the adult, this is a rare trauma mechanism for the intestine, but well known for blunt abdominal trauma in children falling over the handlebars of a bicycle. Most injuries are due to compression and contusion with consecutive ischemia of the intestinal wall and secondary perforation.

In the literature, few case reports of blunt bowel perforation are described.

One case described an atypical colonic rupture due to fall on the back. The first examination was unremarkable, as well as the ultrasound. After a few hours and increasing pain, a CT scan was performed with the diagnosis of the bowel perforation and subsequent surgical therapy [3].

Another case described a patient with blunt abdominal trauma and mesenteric hematoma, which was managed conservatively. After 6 weeks, the patient was re-admitted to the hospital due to abdominal pain. The explorative laparotomy showed a bowel perforation with a mesenteric hematoma. In this case, mesenteric injury caused bowel ischemia with subsequent perforation [4].

We recommend performing a CT scan in patients with blunt abdominal trauma and conspicuous clinical examination. If free air is seen and the patient shows additional clinical signs as increased white blood cells count, abdominal rebound tenderness, fractured ribs or free fluid, surgical intervention might be indicated. In patients with mesenteric hematoma, a surgical exploration is also warranted to rule out bowel ischemia or perforation. If possible, the operation should be performed laparoscopically. If an anastomosis was necessary, we recommend the placement of drains to detect a possible leak early. In case of mesenteric hematoma, a close follow up of the patient is advised, as late perforations can occur.

In trauma patients, it is important to consider bowel perforation, even if the patients only suffered a low-velocity trauma. If a perforation remains undetected or diagnosis is delayed, the morbidity and mortality could increase as a consequence. In our case, the early surgical approach prevented the patient from suffering chemical peritonitis and consecutive septic shock.

## CONFLICT OF INTEREST STATEMENT

None declared.

## FUNDING

None.
